# Novel Method for Processing the Dynamic Calibration Signal of Pressure Sensor

**DOI:** 10.3390/s150717748

**Published:** 2015-07-21

**Authors:** Zhongyu Wang, Qiang Li, Zhuoran Wang, Hu Yan

**Affiliations:** 1 School of Instrument Science and Opto-Electronic Engineering, Beijing University of Aeronautics and Astronautics, Beijing 100191, China; E-Mails: mewan@buaa.edu.cn (Z.W.); zhuoran.cynthia@163.com (Z.W.); 2 Beijing Changcheng Institute of Metrology and Measurement, Beijing 100095, China; E-Mail: yanhuyanxin@126.com

**Keywords:** dynamic calibration, signal processing, strong disturbance, pressure sensor

## Abstract

Dynamic calibration is one of the important ways to acquire the dynamic performance parameters of a pressure sensor. This research focuses on the processing method for the output of calibrated pressure sensor, and mainly attempts to solve the problem of extracting the true information of step response under strong interference noise. A dynamic calibration system based on a shock tube is established to excite the time-domain response signal of a calibrated pressure sensor. A key processing on difference modeling is applied for the obtained signal, and several generating sequences are established. A fusion process for the generating sequences is then undertaken, and the true information of the step response of the calibrated pressure sensor can be obtained. Finally, by implementing the common QR decomposition method to deal with the true information, a dynamic model characterizing the dynamic performance of the calibrated pressure sensor is established. A typical pressure sensor was used to perform calibration tests and a frequency-domain experiment for the sensor was also conducted. Results show that the proposed method could effectively filter strong interference noise in the output of the sensor and the corresponding dynamic model could effectively characterize the dynamic performance of the pressure sensor.

## 1. Introduction

Pressure sensors have been widely used to measure dynamic pressure signals in intelligent control, wind tunnel test, flight test, and so on [1]. The dynamic performance parameters of a pressure sensor are the important indicators of its dynamic measurement capability and are also the main basis to judge whether the needs of a dynamic pressure measurement are met [1,2].

Rapid on-off valves [3], sinusoidal pressure generator [4], and shock tube [3,5] are the most common used dynamic calibration methods. The rising time of rapid on-off valves is about 20 μs [3], which makes it impossible for the pressure sensor whose dynamic response is high. The calibrating pressure produced by a sinusoidal pressure generator can only range from 0 MPa to 3 MPa [3]. Moreover, the degree of waveform distortion of the sinusoidal pressure generator increases greatly in high calibrating frequency. These characteristics make the sinusoidal pressure generator only possible for dynamic calibration with low frequency and small-scale pressure sensor [3]. At present, the rising time of step pressure produced by a shock tube can be controlled less than 0.1 μs, which can be regarded as the standard input signal of dynamic calibration. The duration of platform pressure of a shock tube is usually more than 10 ms, which can fully meet the calibration requirement of most pressure sensors for the duration. Moreover, the calibrating frequency of a shock tube ranges from 2 KHz to 1 MHz, but the natural frequencies of most pressure sensors are less than 1 MHz and the dynamic responses of those pressure sensors are perfect when the frequency is lower than 2 KHz [3]. The above three characteristics make the shock tube widely used in the field of dynamic calibration of pressure sensor.

At present, dynamic calibration using a shock tube is an important way to obtain the dynamic performance parameters of a pressure sensor. Since the shock tube was created in 20th century, it has been widely used in a variety of dynamic experiments. A shock tube can produce a high-speed shock wave and a subsequent step pressure, making it ideal dynamic equipment for calibrating pressure sensors [1]. In 2002, the Instrument Society of America published an important work titled “A Guide for the Dynamic Calibration of Pressure Transducers”, which considered shock tube calibration as the most important calibration methods [2]. The Chinese military standard reference, “Dynamic Calibration Regulation of Pressure Sensors” published in 2005, details the shock tube calibration method [6].

The pressing problem existing in various types of shock tube calibration systems is their frequent subject to the interferences of various noises in different fields, such as reflected shock waves, rarefaction waves, impact of cracked aluminum sheet, tunnel effect of shock tube, vibration noise, and so on. Moreover, the corresponding interference noises contained in the output signal exhibit strong randomness, strong power, unknown distribution, and lack of information and certainty. At times, the frequencies of some interference noises are within the frequency range of step pressure. The effects of these strong interferences create the amplitude variation of the output of calibrated pressure sensors along with considerable randomness, hence the difficulty of directly applying the output signal to perform modeling by the common QR decomposition method [3]. At present, only a few dynamic performance parameters, such as rise time, setting time, resonance frequency, can be directly found from the output signal of a calibrated pressure sensor subjected to a shock tube calibration test. To analyze the signal processing method for removing interferences and to find the true information of the step response of the calibrated pressure sensor are the key points determining the dynamic performance parameters of a calibrated pressure sensor.

Many scholars devote themselves to the study of eliminating interference noise from the output signal of sensor. Ansari [7] presented a wavelet multi-resolution analysis method to separate interference noise from the dynamic pressure signal. The method performed well in the local time-domain, and the multi-scale analysis of the vibration signal was performed. At the unknown probability distribution of the interference noise, the numbers of the wavelet decomposition were selected randomly, resulting in the large uncertainty in the end result. Wu [8] proposed a time-frequency peak filtering method that relies slightly on the filter-window. This method performed well in processing random noise. Liu [9] forwarded a time-varying window mid-value filtering method that realized the most balanced separation for the different strengths of the interference signal. However, the mid-value-filter mainly aimed to process impulse noise, and the ideal performance cannot be obtained when the distribution of the interference noise is unclear or non-additive. Based on the Kalman method, Chen [10] proposed a self-adaptive filter algorithm to deal with the dynamic calibration signal of sensors. Only the errors caused by mechanical installation can be removed. Zhang [11] provided a special whitening filter method to deal with the dynamic calibration signal of pressure sensors excited by the shock tube. It is proved that the square sum of errors between model output and observed output data is the minimum for the calibration system when the input noise is small and the output measurement noise approximates to white noise.

The typical characteristic of the previously mentioned filtering methods is that they all belong to the analysis method of statistical theory. If the distributions of interference noises are completely unknown or the power and randomness of the interference noises are very strong, these methods do not usually apply. Fortunately, a new type of signal processing method, called poor information processing method, has been proposed in recent years. This method is mainly based on the grey system theory, fuzzy set theory, norm method, and rough set theory, and can work well in case of the distributions of unknown interference noises, especially when the samples of the output are very large and the information about the noises is poor. Meng [12] used this type method in the field of surface wave information evaluation where the information about surface roughness was completely unknown. The filtering effect was established more than the classical Gauss method. Wang [13] applied this type of method in the signal processing of sensors used in seismic surveys in which many strong interference noises occurred, and this method solved the problem of extracting true information from a noisy output. These poor information processing methods lay the foundation for processing the dynamic calibration signal of a pressure sensor in shock tube calibration test.

In addition, Xu [14,15,16,17,18] focused on the filtering problem for the measured signal containing strong vibration signal noise. He forwarded a method for extracting the true information from the sensor’s output signal. Moreover, an evaluation for the filtering performance was performed from the point of frequency-domain. His work provided a reference for the present research.

A dynamic calibration system is presented in this paper. The key is to examine the filtering processing method for the output signal of the pressure sensor calibrated under the present system. Key processing on difference modeling is applied for the obtained signal to find several generating sequences. Fusion processing is performed to process the obtained generating sequences. Finally, by implementing the common QR decomposition method for the extracted true information, a dynamic model characterizing the dynamic performance of the calibrated pressure sensor is established. Calibration test and frequency experiment are separately performed to verify the effectiveness of the system and of the processing algorithm.

## 2. Hardware Description of the Shock Tube Calibration System

**Figure 1:**
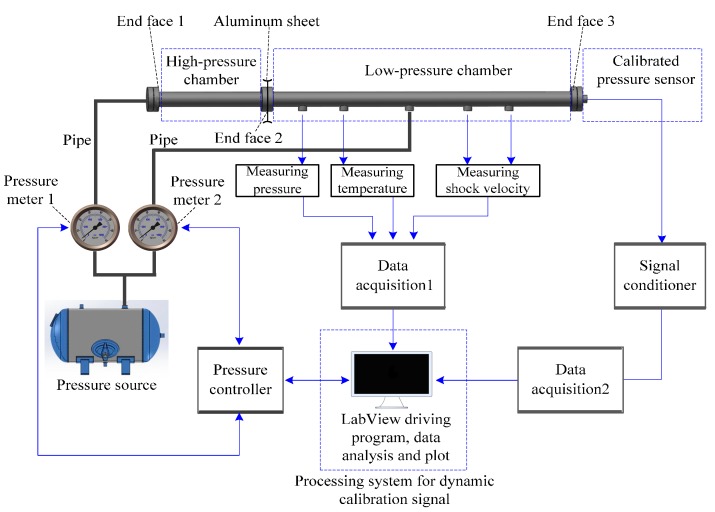
Schematic diagram of the shock tube calibration system.

The hardware of the shock tube dynamic calibration system for pressure sensors is shown in Figure 1. The hardware comprises a shock tube, a pressure meter, a pressure controller, a temperature meter, a velocity meter, a signal conditioner, two data acquisitions and a processing system. The calibrated pressure sensor is installed at the end face 3. The processing system includes a personal computer, the driving program of the present processing algorithm by LabView, and a man-machine interface module. Meters are used to measure the data of measurable interference factors. For example, the temperature meter measures the increase in temperature of a low-pressure chamber during calibration test. The temperature increase can usually reach up to 300 °C, which creates significant interference for the output of the calibrated pressure sensor. Based on these measured data, the effects of some interference noises can be removed directly from the sensor’s output.

The high-pressure and low-pressure chambers are separated by an aluminum sheet. In the calibration experiment, the two chambers are filled with different pressures of nitrogen. As the pressure difference, which can be controlled by adjusting the pressure meters 1 and 2, reaches a particular degree, the aluminum sheet is punctured. The expanding gas in the high-pressure chamber fleetly rushes into the low-pressure chamber, and a shock wave is almost simultaneously produced. In the process, a pressure jump is formed before the shock wave, and the step pressure is produced. The step pressure arrives at the end face 3 and acts on the calibrated pressure sensor.

During the calibration experiment, interference noises also act on the calibrated pressure sensor, in the form of a tunnel effect of the shock tube, a mechanical vibration noise of the shock tube, a reflected shock wave from end faces 1 and 2, and a rarefaction wave from end faces 1 and 2. Moreover, the fragments from the punctured aluminum sheet may hit the calibrated pressure sensor installed in end face 3 and lead to strong interference noise. The strength of the interference noise is usually much more than the strength of the step pressure signal and leads to the output waveform of the calibrated pressure sensor increase exceedingly during the period of increase. In addition, the profile of the punctured aluminum sheet is usually irregular. When the shock wave occurs, a reflected shock wave is formed and then acts on the calibrated pressure sensor.

The calibrated pressure sensor transforms both the step signal and interference noises to an electrical signal. The output of the calibrated pressure sensor is processed by signal conditioner and data acquisition 2, successively. The sampled data are finally processed by the processing system using the present processing algorithm. Processing results are displayed in the LCD of the personal computer containing the man-machine interface module.

In sum, the effects of interference noises on the step signal are considerably large and lead to a high amount of random noises in the output of the calibrated pressure sensor. These random noises can even cause serious distortion of the output signal. In this case, building a dynamic model by using the signal directly is infeasible, and the obtained model cannot specifically characterize the dynamic characteristics of the calibrated pressure sensor.

## 3. Dynamic Calibration Signal Processing

### 3.1. Method Principle

**Figure 2:**
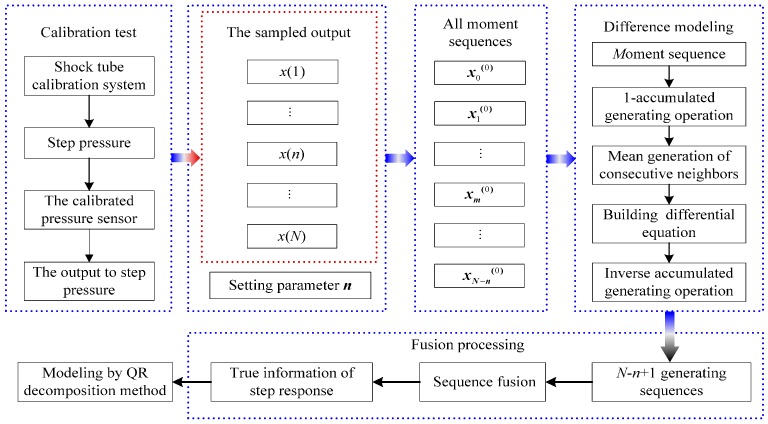
Principle for processing the dynamic calibration signal.

The processing principle for the dynamic calibration signal of the pressure sensor in the shock tube calibration test is briefly described in Figure 2. As can be seen, the parameters *x*(1), …, *x*(*n*), …, *x*(*N*) represent the points of the sampled output of calibrated pressure sensor, and *N* represents the total member of sampled output; the parameter *n* represents the length of zero moment sequence and can be set by the user; *x*_0_^(0)^ represents the zero moment sequence, *x_m_*^(0)^ represents the *m* moment sequence and *x_N−n_*^(0)^ represents the *N* − *n* moment sequence. The detailed process is expressed in the succeeding sections.

### 3.2. Processing Algorithm

Theoretically speaking, the output of the calibrated pressure sensor in the shock tube calibration test should be a standard step signal. Unfortunately, this signal is often subject to various field interferences, such as the tunnel effect, mechanical vibration noise, reflected shock waves, rarefaction waves, and the impact of the fragments from the aluminum sheet. These interferences often generate a strong disturbance in the calibrated pressure sensor and cause strong random noises in the sensor’s output. Supposing that the output is *x*(*t*), this output is composed of step response *s*(*t*), impact random noise *i*(*t*), and vibration random noise *v*(*t*). The output *x*(*t*) can be expressed as

(1) x(t)=s(t)+i(t)+v(t)

Using the data acquisition to capture the output *x*(*t*), the sampled output, namely, the original sequence, is obtained and can be given by Equation (2).

(2) x={x(1),x(2),⋯,x(n),⋯,x(N)}

The former *n* data of the sampled output *x* is used as zero moment sequence, which can be expressed as ${x_{0}}^{\left(0\right)}=\{x(1),x(2),\cdots ,x(n)\}$, where *n* is the length of the sequence and *n* is much lesser than *N*. The sequence of 1-accumulated generating operation [19,20] can be obtained by

(3) $${x_{0}}^{\left(1\right)}=\{{x_{0}}^{\left(1\right)}(1),{x_{0}}^{\left(1\right)}(2),\cdots ,{x_{0}}^{\left(1\right)}(n)\}$$

where ${x_{0}}^{\left(1\right)}(k)=\sum_{i=1}^{k}{x_{0}}^{\left(0\right)}(i)$, *k* = 1, 2,…, *n*.

The MEAN data sequence of *x*_0_^(1)^ is described by

(4) $${z_{0}}^{\left(1\right)}=\{{z_{0}}^{\left(1\right)}(2),{z_{0}}^{\left(1\right)}(3),\cdots {z_{0}}^{\left(1\right)}(k),\cdots {z_{0}}^{\left(1\right)}(n)\}$$

where ${z_{0}}^{\left(1\right)}(k)=\frac{1}{2}\left({x_{0}}^{\left(1\right)}(k)+{x_{0}}^{\left(1\right)}(k-1)\right)$, *k* = 2, 3,…, *n*.

Based on the members of the zero moment sequence and of the mean data sequence, a differential equation concerning the sequence *x*_0_^(0)^ is established.

(5) $${x_{0}}^{\left(0\right)}(k)+a_{0}{z_{0}}^{\left(1\right)}(k)=b_{0}$$

where *a*_0_ and *b*_0_ are the undetermined coefficients.

The least squares solution to Equation (5) can be derived by

(6) $${{\hat{x}}_{0}}^{\left(1\right)}(k)=({x_{0}}^{\left(0\right)}(1)-\frac{b_{0}}{a_{0}})e^{-a_{0}(k-1)}+\frac{b_{0}}{a_{0}}$$

where *k* = 1, 2,…, *n*.

After the inverse accumulated generating operation [19,20] (IAGO), a model generating the value corresponding to the sequence *x*_0_^(0)^ can be obtained by

(7) $${{\hat{x}}_{0}}^{\left(0\right)}(k+1)={{\hat{x}}_{0}}^{\left(1\right)}(k+1)-{{\hat{x}}_{0}}^{\left(1\right)}(k)$$

where *k* = 1, 2,…, *n*.

Similarly, the data sequence at *m* moment (*i.e.*, *m* moment sequence) is

(8) $${x_{m}}^{\left(0\right)}=\{{x_{m}}^{\left(0\right)}(1),{x_{m}}^{\left(0\right)}(2),\cdots ,{x_{m}}^{\left(0\right)}(n)\}=\{x^{\left(0\right)}(m+1),x^{\left(0\right)}(m+2),\cdots ,x^{\left(0\right)}(m+n)\}$$

where *m* = 0, 1,…, *N* − *n*.

The solution corresponding to the differential equation of sequence *x_m_*^(0)^ is given by

(9) $${{\hat{x}}_{m}}^{\left(1\right)}(k)=({x_{m}}^{\left(0\right)}(1)-\frac{b_{m}}{a_{m}})e^{-a_{m}(k-1)}+\frac{b_{m}}{a_{m}}$$

where *k* = 1, 2,…, *n*.

The solution corresponding to sequence *x_m_*^(0)^ can be obtained by IAGO.

(10) $${{\hat{x}}_{m}}^{\left(0\right)}(k+1)={{\hat{x}}_{m}}^{\left(1\right)}(k+1)-{{\hat{x}}_{m}}^{\left(1\right)}(k)$$

where *k* = 1, 2,…, *n*.

Based on the result sets of Equation (10), a generating sequence corresponding to the *m* moment can be obtained. In the entire process of deriving the sampled output *x*, *N* − *n* + l generating sequences are obtained. With the increasing value of parameter *m*, the generating sequence curves, constructed by the generating sequences, slide along the profile of the sequence curve constructed by the sampled output *x*. For the *i*th sampling data of sampled output *x*, *T_i_* generating sequence curves can be used to describe them.

**Figure 3:**
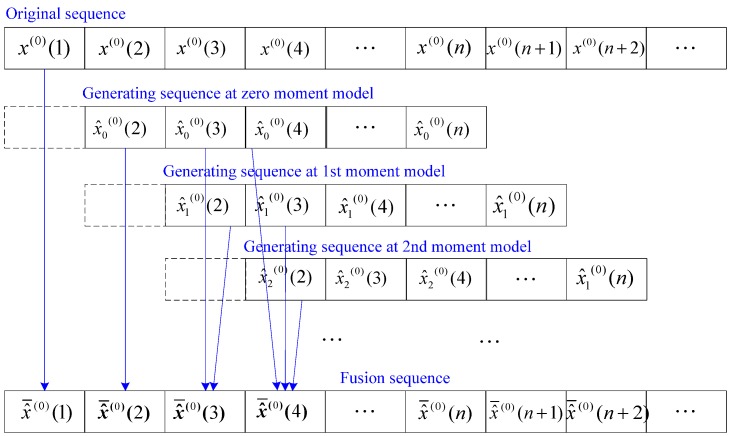
Fusion processing for generating sequences.

Supposing the weights of each curve are equal to each other, the average value of all curves at the same point is regarded as the true value of the output of the pressure sensor at the moment. By the fusion processing, all average values construct a fusion sequence that represents the true information of the step response of the calibrated pressure sensor. The fusion processing is also called sequence fusion, and its principle is illustrated in Figure 3. The corresponding fusion sequence, namely, the true information of step response, is given by Equation (11).

(11) $${\bar{\hat{x}}}^{\left(0\right)}(i)=\{\begin{array}{ll} \frac{1}{T_{i}}\sum_{m=0}^{T_{i}-1}{\hat{x}}_{m}(i-m), & 1\leq i<n\\ \frac{1}{T_{i}}\sum_{m=i-n}^{i-n+T_{i}-1}{\hat{x}}_{m}(i-m), & n\leq i\leq N \end{array}$$

in which

(12) $$T_{i}=\{\begin{array}{cc} 1, & i=1\\ i-1, & 1<i\leq n\\ n-1, & n<i\leq N-n+2\\ N\text{-}i+1 & N-n+2<i\leq N \end{array}$$

where *i* is the serial number of the fusion sequence and *T_i_* is the fusion number, respectively.

Finally, the common QR decomposition method [3] is applied for the fusion sequence ${\bar{\hat{x}}}^{\left(0\right)}$, and a dynamic model representing the dynamic performance of calibrated pressure sensor is found.

## 4. Experiments

A shock tube calibration test is performed to verify the effectiveness of the system and of the processing algorithm.

### 4.1. Experimental Results of the Shock Tube Calibration

**Figure 4:**
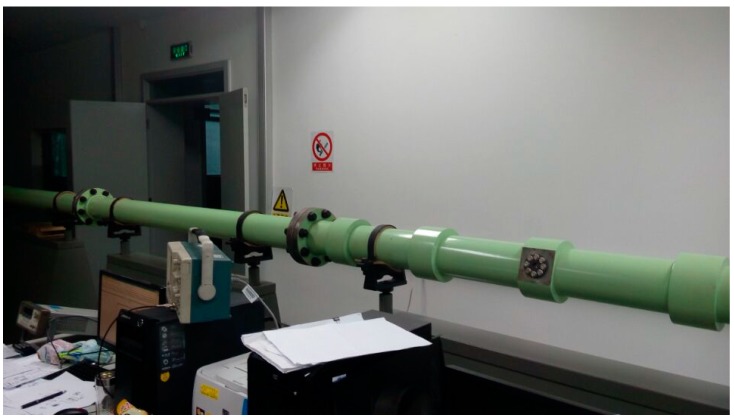
Shock tube calibration test.

The test is conducted at the China Aerospace Test Center (CATC). The piezoresistive pressure sensor T24956, Endevco 8530C-15 series type, is used as the calibrated object. The thickness of the aluminum sheet is about 0.07 mm. The sampling frequency of data acquisition 2 is 5,000,000 Hz. The experiment site is shown in Figure 4.

**Figure 5:**
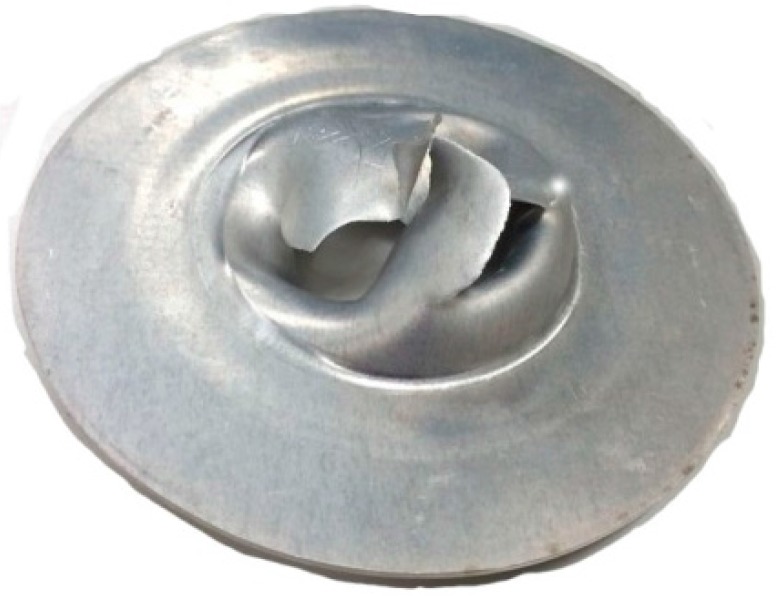
Punctured aluminum sheet.

Figure 5 shows the punctured aluminum sheet used in the shock tube calibration test. As shown in the figure, the aluminum sheet is punctured incompletely.

**Figure 6:**
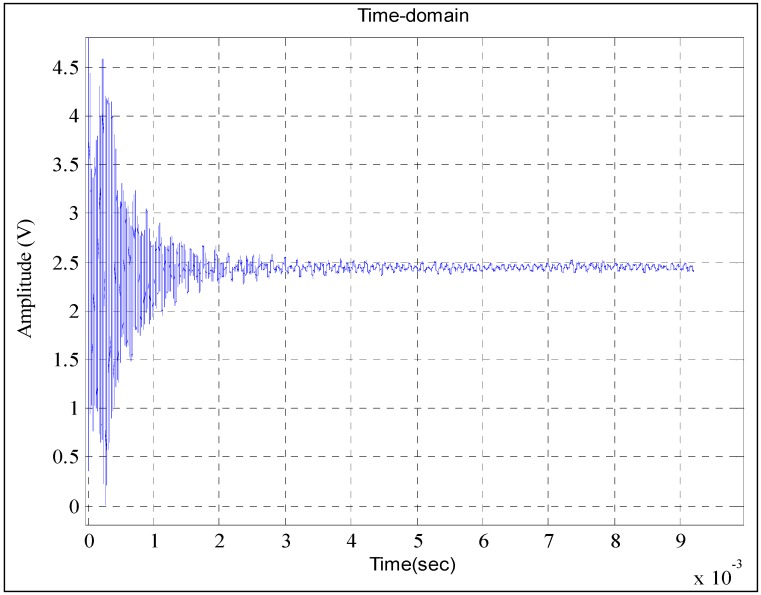
Output signal of the calibrated pressure sensor in the shock tube test.

The time-domain waveform of the output of the calibrated pressure sensor is shown in Figure 6. As can be seen, the response waveform to the step pressure is highly disorganized, especially at the state of 0–1 × 10^−3^ s where the fluctuations of the time-domain waveform are exceedingly large and can be as much as 2.5 V. Hence, a smooth curve, which is absolutely essential to build the dynamic model for a calibrated pressure sensor, is impossible to identify.

As shown in Figure 6, during the period of increase, the waveform of output signal exists in a short-term fluctuation. Thus, the fragments from the punctured aluminum sheet hit the calibrated pressure sensor. The operations on difference modeling and fusion processing are then successively adopted to process the output signal of the calibrated pressure sensor T24956.

**Figure 7:**
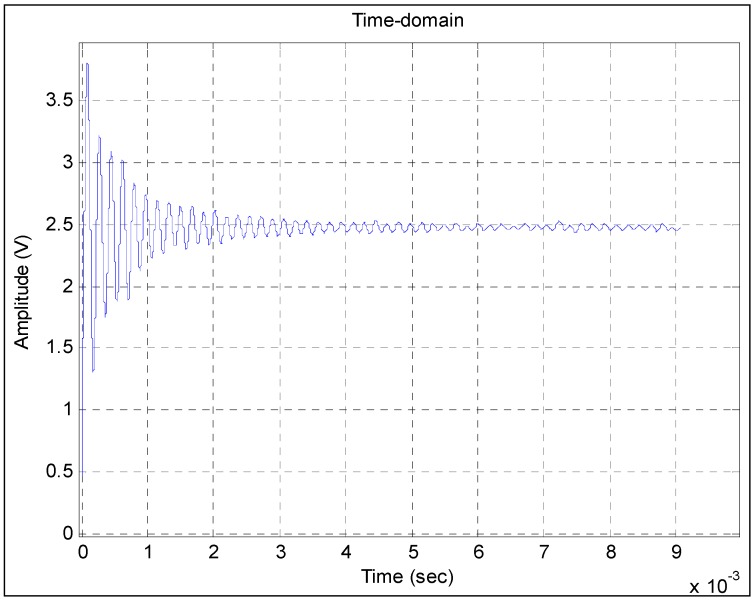
Filtering result obtained by the presented method in this study.

Figure 7 shows the filtered time-domain waveform of the output signal of the calibrated pressure sensor. From the figure, we can easily identify a smooth curve that is the true information of the step response of the calibrated pressure sensor. Obtaining the true information of the step response is the foundation of the modeling method, similar to the QR decomposition method. The performance of the obtained model is closely related to the filtering result. In Figure 7, the smoothness of the filtered time-domain waveform indicates that most of the strong interferences, such as impact and vibration random noises, have been filtered out.

**Figure 8:**
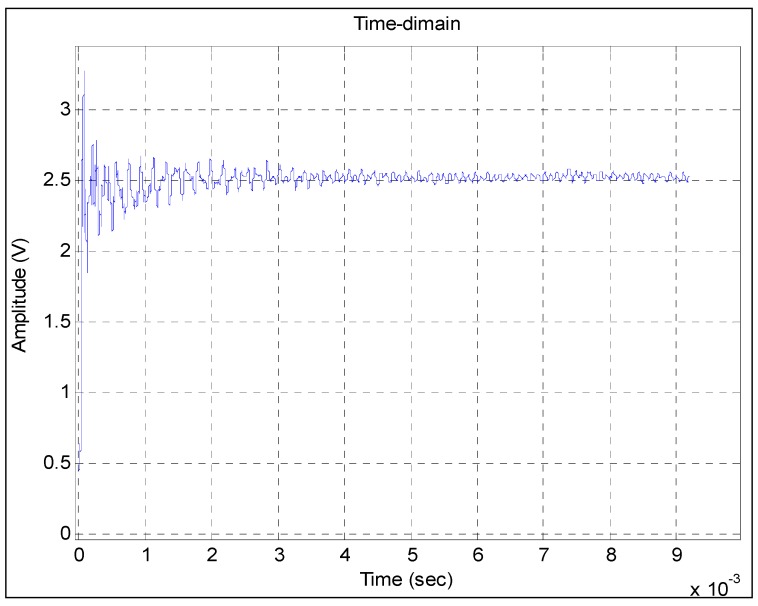
Filtering result obtained by the Kalman method.

Kalman method [10,21] is a common method implemented to process the output signal of the pressure sensor and can be considered as a comparing method in this paper. The curve shown in Figure 8 is the filtering result of the Kalman method. Compared with the time-domain waveform shown in Figure 6, a curve is hardly identified. The unsmooth curve indicates some interference in the filtering result. The remaining interference can have a major adverse effect on the accuracy of the dynamic model.

By implementing the common QR decomposition method for the filtering result of presented method in this study, a dynamic model characterizing the dynamic characteristics of the calibrated pressure sensor is found and is expressed as follows. For convenience, this dynamic model is referred to as Model 1 in the succeeding sections.

(13) $$G1=\frac{1.2603\times 10^{9}}{s^{2}+3470.79s+1.2603\times 10^{9}}$$

where s is the Laplace operator.

Similarly, we also perform the QR decomposition method for the filtering result of the Kalman method, and a model, shown as follows, is obtained. This model is referred to as Model 2 in the succeeding sections.

(14) $$G2=\frac{0.01s^{2}-7.35\times 10^{3}s+1.8\times 10^{9}}{s^{2}+1.0456\times 10^{4}s+1.16\times 10^{9}}$$

where s is also the Laplace operator.

**Figure 9:**
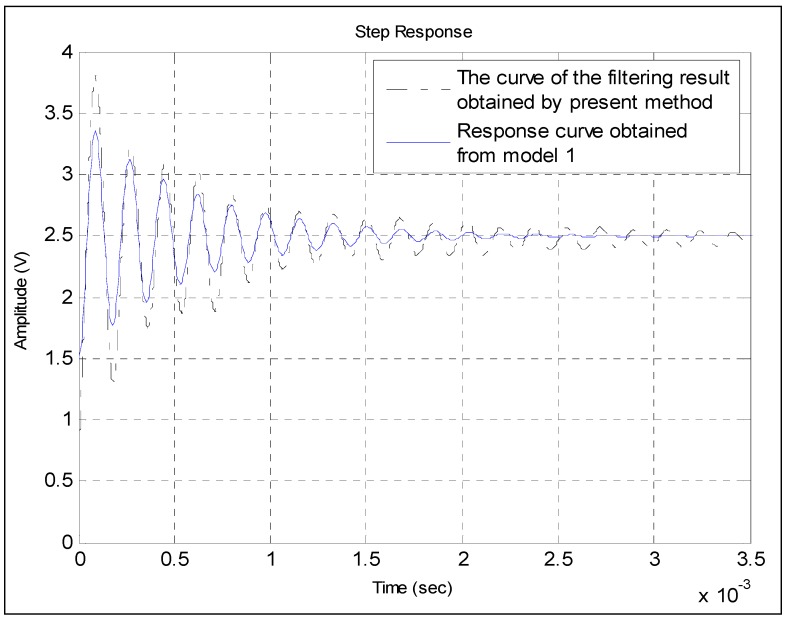
Comparison between the step response obtained from Model 1 and the curve of the filtering result obtained by the presented method.

Figure 9 shows the response comparison of the calibrated pressure sensor. The blue solid line represents the step response obtained from Model 1. As shown in the figure, the waveforms of the two curves are very similar to each other.

**Figure 10:**
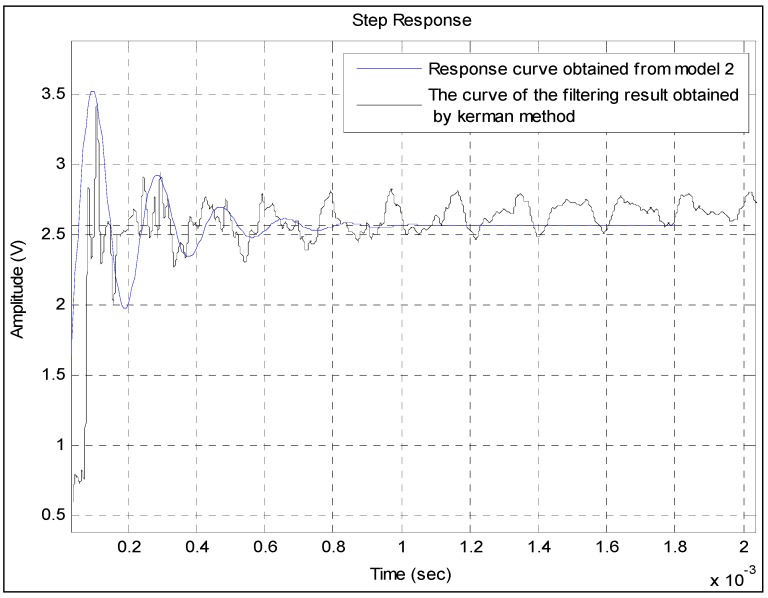
Comparison between the step response obtained from Model 2 and the curve of the filtering result obtained by the Kalman method.

The blue response curve shown in Figure 10 is obtained from Model 2. Although the random fluctuation of the filtering result curve of the Kalman method is very large, the waveform of the blue response curve is basically consistent with that of the curve. Therefore, the obtained model basically reflects the information of the filtering results. The two curves do not fully overlap because of the remaining interference noises. The comparison also shows that the power of the interference noises is larger than that of the step pressure from 0.7 × 10^−3^ s to 2 × 10^−3^ s. This finding is because the frequencies of some interference noises are just within the frequency range of the step pressure. Over time, the power of some interference noises remains strong, while that of step pressure becomes weak. In this case, the common filter methods, including the Kalman method, cannot be used to eliminate them. By contrast, in Figure 9, the curve of the filtering result obtained by the presented method is rather smooth, which indicates that most of the interference noises have been filtered out. Therefore, the waveforms of the two curves are very similar to each other.

### 4.2. Experimental Results of the Frequency-Domain Experiment

**Figure 11:**
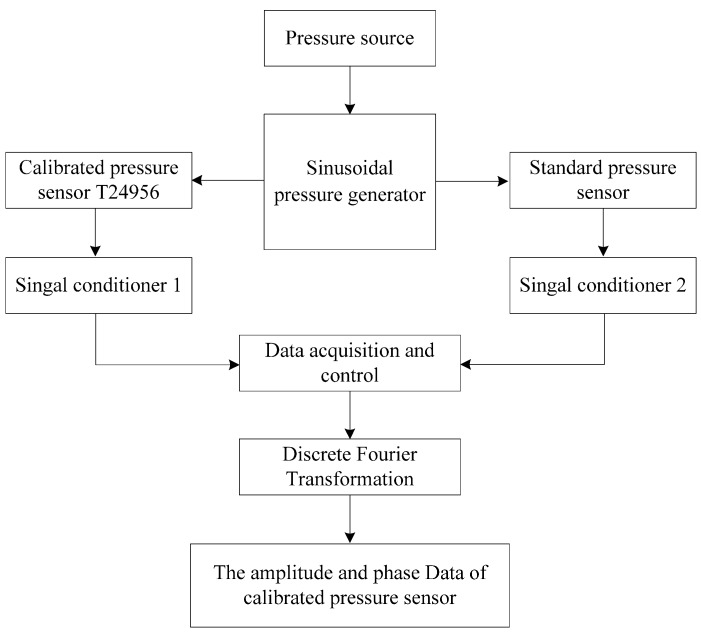
Schematic diagram of the frequency-domain experiment.

The frequency-domain experiment is performed to verify the reliability of the obtained models. The experimental set-up consists of a pressure source, a sinusoidal pressure generator, the calibrated pressure sensor T24956, a standard pressure sensor, two signal conditioners, a high-speed data acquisition card, and a personal computer. Process control and data processing are performed by the personal computer. The schematic diagram of the frequency-domain experiment is shown in Figure 11.

The frequency-domain experiment adopts the comparison principle. In the experiment, the pressure sensor T24956 and a standard pressure sensor are used to simultaneously measure the excitation signal produced by a sinusoidal pressure generator. Using a specific algorithm, the amplitude and phase parameters measured by pressure sensors are determined. By comparing the parameters measured by the standard pressure sensor with those measured by the pressure sensor T24956, the amplitude frequency and phase frequency characteristics of the pressure sensor T24956 are obtained. The experimental procedure is as follows.

(1) A sinusoidal pressure generator is recognized to simultaneously excite two pressure sensors at frequency *f_i_* (*i* = 1, 2,…, *n*). Meanwhile, the outputs of the two pressure sensors are separately processed by signal conditioners, with the output of the standard pressure sensor as the true information of the generated sinusoidal pressure. The processed signals are then collected by data acquisition, acquiring two discrete voltage sequences.
(2) Discrete Fourier Transformation is applied to separately handle the two voltage sequences, and the amplitude *A_fi_*_1_ (*i* = 1, 2,…, *n*) of the sinusoidal pressure produced in the experiment and the corresponding phase θ*_fi_*_1_ (*i* = 1, 2,…, *n*) are found. Similarly, the amplitude *A_fi_*_2_ (*i* = 1, 2,…, *n*) and the corresponding phase θ*_fi_*_2_ (*i* = 1, 2,…, *n*) measured using the pressure sensor T24956 for the sinusoidal pressure are obtained.
(3) Comparing the amplitude and phase measured by standard pressure sensor with those measured by the pressure sensor T24956 at frequency *f_i_* (*i* = 1, 2,…, *n*), the phase shift and amplitude sensitivity error of pressure sensor T24956 are determined. The accurate frequency characteristic of the pressure sensor T24956 is consequently found.

**Figure 12:**
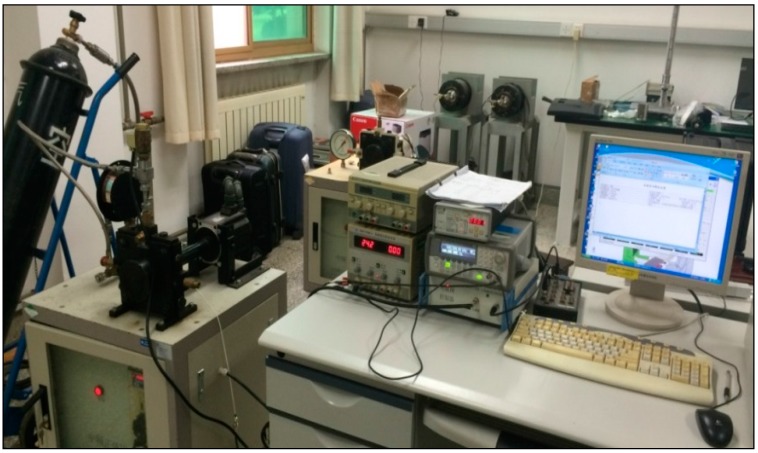
Experimental environment of the frequency-domain experiment.

The experimental environment set up is shown in Figure 12. The key parameters of the experimental equipment are shown in Table 1. A number of considerations are taken to ensure that the indices of the sinusoidal pressure generator can fully meet the experimental requirements of pressure sensor T24956, and the indices of the standard pressure sensor are completely superior to those of the pressure sensor T24956.

**Table 1:** Key parameters of the frequency-domain experiment.

Indices of Sinusoidal Pressure Generator		Parameters	Indices of the Standard Pressure Sensor	Parameters
Calibrated pressure range		0–5 MPa	Amplitude sensitivity error [6]	±2 dB
Operating frequency range		0.1–120,000 Hz	Measurement uncertainty of phase angle [6]	±0.5°
Degree of waveform distortion [3]	0.1–10,000 Hz	0.5%–1%	Measurement uncertainty of amplitude sensitivity [6]	<1%
	10,000–30,000 Hz	1%–2%		
	30,000–70,000 Hz	2%–4%	Static accuracy level [6]	1 grades
	70,000–120,000 Hz	4%–7%		

**Figure 13:**
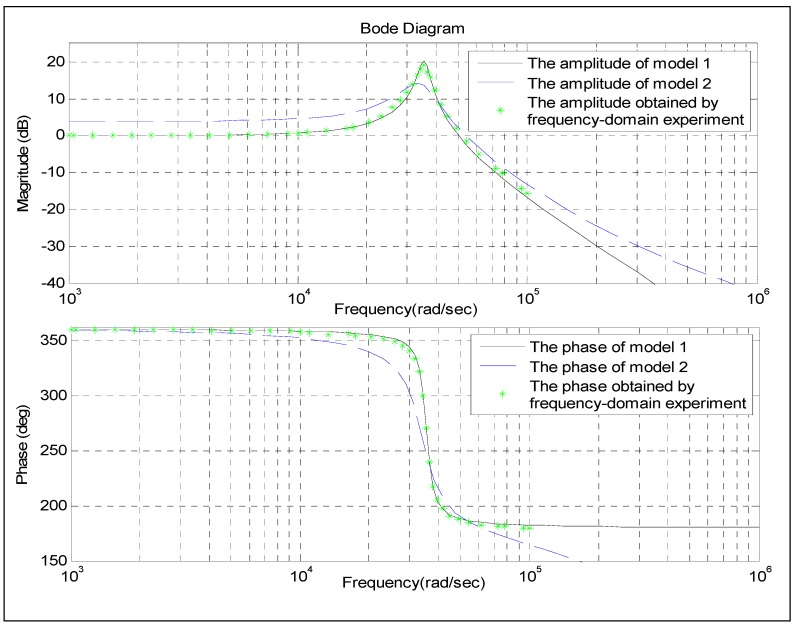
Frequency-domain characteristic data obtained by different methods.

The characteristic data of pressure sensor T24956 obtained by the frequency-domain experiment are shown in Figure 13 in the * notation. The Bode Diagram (Figure 13) can be obtained using the two models. The amplitude and phase frequency curves from Model 1 nearly overlap with those obtained by the frequency-domain experiment (Figure 13), especially, when the frequency is lower than 10^4^ rad/s. When the frequency increases gradually from 10^4^ rad/s, the differences among the same curve types increase slightly. By contrast, large differences are evident among the same curve types are obtained by the frequency-domain experiment and from Model 2. The above analysis results show that the performance of Model 1 is better than that of Model 2. We will perform a quantitative analysis for these differences later on.

### 4.3. Discussion

**Figure 14:**
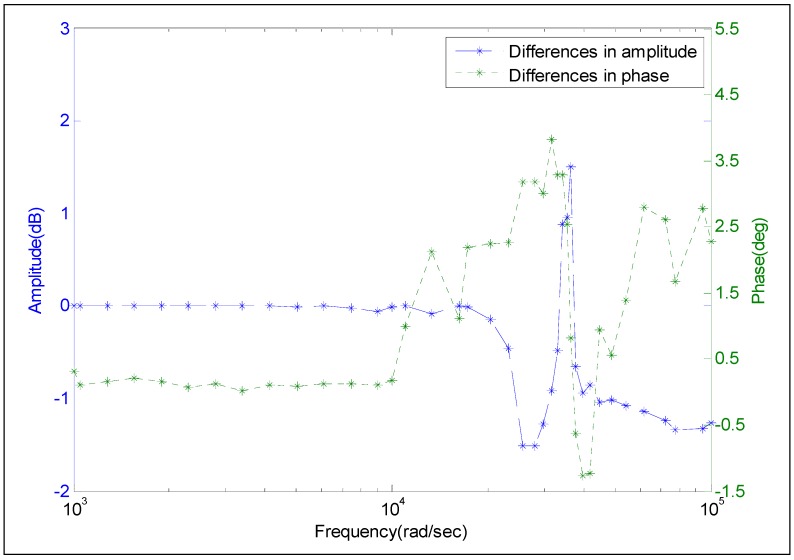
Differences in the amplitude-frequency and phase-frequency between the frequency-domain experiment and Model 1.

The blue curve in Figure 14 illustrates the differences between the amplitude of Model 1 and that obtained by the frequency-domain experiment. The green curve represents the difference between the phase of Model 1 and that obtained by the frequency-domain experiment. When the frequency is lower than 10^4^ rad/s, the differences in amplitude are within [−0.1, 0.1] dB and the differences on phase are within [−0.3, 0.3] deg. These differences, whether on amplitude or phase, are rather small and can be neglected. Moreover, the working frequency scope of pressure sensor T24956 is completely contained in [0, 10^4^] rad/s. This frequency scope is within the frequencies considered in engineering for pressure sensors. Although the differences in both amplitude and phase are increasing progressively at a high frequency scope, they still remain acceptable. Thus, the frequency characteristics obtained from Model 1 are proved to be acceptable.

**Figure 15:**
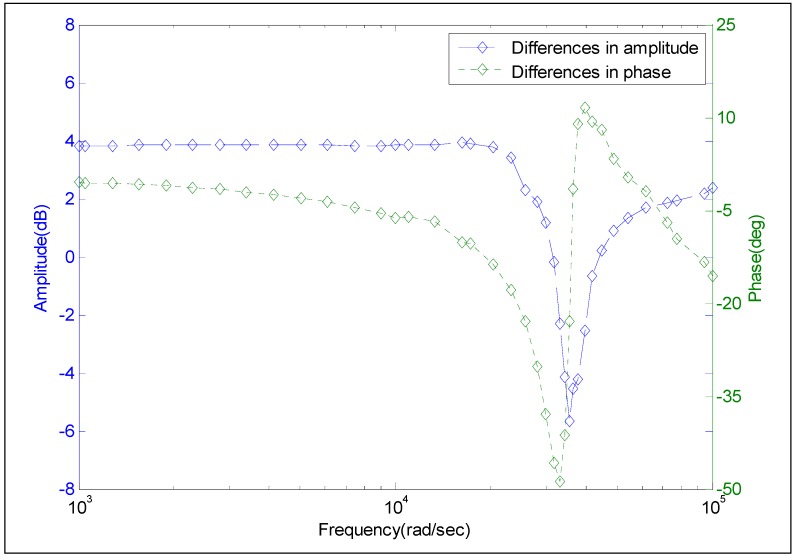
Differences in the amplitude-frequency and phase-frequency between the frequency-domain experiment and Model 2.

The blue curve in Figure 15 represents the differences between the amplitude of Model 2 and that obtained by the frequency-domain experiment. The green curve represents the difference between the phase of Model 2 and that obtained by the frequency-domain experiment. The difference in amplitude is within the range of [3, 5] dB, which is much larger than that in Figure 14. The difference in phase is in the range of [−3, 7] deg which is also much larger than the corresponding data in Figure 14. As can be seen in Figure 15, at the frequency higher than 10^4^ rad/s, the differences in amplitude or in phase considerably increase as frequency increases. These differences can reach −6 dB and −50°, separately, which are regarded to be unacceptable. The analysis results based on Figure 15 show that Model 2 could not characterize the dynamic performance of the calibrated pressure sensor T24956.

Based on the comparative results, the acceptability of Model 1 indicates that the proposed processing algorithm is more advantageous than the current method in processing the dynamic calibration signal of the pressure sensor.

## 5. Conclusions

A dynamic calibration system based on the shock tube is presented to find the dynamic performance parameters of the calibrated pressure sensor. Based on the features of the output signal of the calibrated pressure sensor and the interference noises observed in the calibration test, a processing algorithm is proposed to obtain the dynamic calibration signal of the pressure sensor and to solve the problem of true information extraction. This algorithm adopts difference modeling and fusion processing to process the output of the calibrated pressure sensor, and then the QR decomposition method is applied to perform modeling by using the obtained true information of the step response.

A pressure sensor T24956 type of Endevco 8530C-15 was used to perform the test under the proposed dynamic calibration system. To verify the effectiveness of the system and processing algorithm, a shock tube calibration test and a frequency-domain experiment were conducted consecutively. The presented method in this study and the Kalman filtering method were implemented to process the output signal. The filtering results were subjected separately to the QR decomposition method. Two dynamic models were obtained and then were solved in the frequency-domain. Moreover, a frequency-domain experiment for the calibrated pressure sensor was carried out. The results of the frequency-domain experiment were compared with the frequency results of two dynamic models. Comparison results show that the frequency data obtained from the model, based on the filtering results of the presented method, were consistent with that of the experiment. In the low-frequency scope, the maximal difference in the phase is not more than 0.1 dB and in amplitude is less than 0.3°, which is much less than those obtained by the experiment and another model. Experiments also show that the proposed processing algorithm can effectively filter strong interference noises in the output signal of the calibrated pressure sensor and that the corresponding model can particularly characterize the dynamic performance of the calibrated pressure sensor. Both the calibration system and the processing algorithm exhibited excellent performances.

Compared with other dynamic calibration methods, such as sinusoidal pressure generator and rapid on-off valves, the shock tube has been widely used in the field of dynamic calibration of pressure sensors. However, because the power and randomness of the interference noises produced in shock tube calibration process is strong, the shock tube is generally only used for the dynamic calibration of low-precision pressure sensor. The present processing algorithm can extract the true information from the calibrated pressure sensor’s output signal, no matter what distributions noises obey and how strong the power and randomness of interference noises is, which makes it possible to use the shock tube in the dynamic calibration of high-precision pressure sensor in practice. Although the parameter *n* of the proposed method may have some impacts on the filtering result, but now the method itself does not give the solution. Further improvement for the present method is to discuss the impacts and determine the optimal value of parameter *n*.
